# Association of transcranial Doppler blood flow velocity slow waves with delayed cerebral ischemia in patients suffering from subarachnoid hemorrhage: a retrospective study

**DOI:** 10.1186/s40635-021-00378-8

**Published:** 2021-03-26

**Authors:** Vasilios E. Papaioannou, Karol P. Budohoski, Michal M. Placek, Zofia Czosnyka, Peter Smielewski, Marek Czosnyka

**Affiliations:** 1 Department of Intensive Care Medicine, Alexandroupolis Hospital, Democritus University of Thrace, 68100 Alexandoupolis, Greece; 2 Academic Neurosurgery Unit, Brain Physics Lab, Addenbrooke’s Hospital, Box 167, Cambridge, CB20QQ UK; 3 Department of Neurosurgery, Cambridge University Hospitals, Cambridge, CB20QQ UK; 4 Department of Biomedical Engineering, Faculty of Fundamental Problems of Technology, Wrocław University of Science and Technology, 50-370 Wrocław, Poland

**Keywords:** Cerebral blood flow, Delayed cerebral ischemia, Subarachnoid hemorrhage, Slow waves, Time constant, Transcranial Doppler, Vasospasm

## Abstract

**Background:**

Cerebral vasospasm (VS) and delayed cerebral ischemia (DCI) constitute major complications following subarachnoid hemorrhage (SAH). A few studies have examined the relationship between different indices of cerebrovascular dynamics with the occurrence of VS. However, their potential association with the development of DCI remains elusive. In this study, we investigated the pattern of changes of different transcranial Doppler (TCD)-derived indices of cerebrovascular dynamics during vasospasm in patients suffering from subarachnoid hemorrhage, dichotomized by the presence of delayed cerebral ischemia.

**Methods:**

A retrospective analysis was performed using recordings from 32 SAH patients, diagnosed with VS. Patients were divided in two groups, depending on development of DCI. Magnitude of slow waves (SWs) of cerebral blood flow velocity (CBFV) was measured. Cerebral autoregulation was estimated using the moving correlation coefficient Mxa. Cerebral arterial time constant (tau) was expressed as the product of resistance and compliance. Complexity of CBFV was estimated through measurement of sample entropy (SampEn).

**Results:**

In the whole population (*N* = 32), magnitude of SWs of ipsilateral to VS side CBFV was higher during vasospasm (4.15 ± 1.55 vs before: 2.86 ± 1.21 cm/s, *p* < 0.001). Ipsilateral SWs of CBFV before VS had higher magnitude in DCI group (*N* = 19, *p* < 0.001) and were strongly predictive of DCI, with area under the curve (AUC) = 0.745 (*p* = 0.02). Vasospasm caused a non-significant shortening of ipsilateral values of tau and increase in SampEn in all patients related to pre-VS measurements, as well as an insignificant increase of Mxa in DCI related to non-DCI group (*N* = 13).

**Conclusions:**

In patients suffering from subarachnoid hemorrhage, TCD-detected VS was associated with higher ipsilateral CBFV SWs, related to pre-VS measurements. Higher CBFV SWs before VS were significantly predictive of delayed cerebral ischemia.

## Background

Cerebral vasospasm (VS) and delayed cerebral ischemia (DCI) constitute major complications following subarachnoid hemorrhage (SAH). DCI has been shown to occur in approximately 40% of patients suffering from SAH. It is more common in those who develop VS in large cerebral arteries, since arterial narrowing has a delayed onset with a peak between 5- and 14 days post-ictus [1]. However, the maximum rate of DCI in patients with VS is around 50%, whereas up to one-third of patients with DCI do not exhibit large artery vasospasm [1, 2]. In this respect, different studies have found that a combination of VS and dysfunction of cerebral autoregulation during the first 4–5 days post-SAH correlate with the occurrence of DCI [3–5]. Such scenario is in accordance with Harper’s dual-insult theory, which states that two hemodynamic insults, such as vascular spasm and autoregulatory failure, are needed to induce ischemia [6]. Nevertheless, such theory cannot explain why hypoperfusion can also be observed in areas not supplied by spastic arteries [7].

Different experimental studies have found that during the acute phase of SAH, global cerebral ischemia, blood–brain barrier disruption, cortical spreading depolarizations, microvascular spasm with endothelial dysfunction, as well as activation of an inflammatory cascade might contribute to increased tissue vulnerability to secondary insults [1, 2, 8]. It is unclear whether disturbed autoregulation is a consequence of such pathophysiological mechanisms. However, as has been suggested [7], loss of autoregulation is an ongoing and dynamic process with occasionally different mechanisms of origin. Thus, macrovascular spasm leads to distal compensatory vasodilatation with shortening of autoregulatory plateau which could signal impaired autoregulation upon testing, whereas microvascular spasm might induce a shift of the plateau to the right, towards higher arterial blood pressure (ABP) [7].

Surrogate markers of cerebral blood flow (CBF), such as transcranial Doppler (TCD) cerebral blood flow velocity (CBFV), are frequently used to monitor development of VS [9], as well as integrity of autoregulation, through estimation of the dynamic changes that take place between ABP and CBFV [10]. Testing of autoregulation requires the observer to apply a hemodynamic stimulus, such as a pharmacologic increase in ABP, increase in arterial pCO_2_, etc., controlling the exact time and grade of stimulation, and synchronously measuring a change in CBF, in order to quantify the reactive autoregulatory forces. However, and despite increased precision of such methods, practical and clinical reasons limit autoregulation testing to infrequent, discontinuous measurements, and these techniques have not been used for continuous monitoring. Continuous methods of autoregulation monitoring rely on the observation of spontaneous responses of CBFV to spontaneous fluctuations in cerebral perfusion pressure (CPP) or ABP. Averaging the repeated measures overtime reduces estimation error and renders the method clinically useful. This couples to the clinical advantage of not requiring potentially harmful hemodynamic stimuli to patients with vulnerable cerebral vasculature [11].

Different studies by examining the relation between CPP/ABP and CBFV have indicated that cerebral autoregulation is a frequency-dependent phenomenon [10–12]. Thus, mechanisms mediating autoregulation in the low-frequency range of ‘slow waves’ (0.005 to 0.05 Hz) may include myogenic, neurogenic and endothelium-derived processes [11, 12]. On the contrary, in the high-frequency range and particularly above 0.2 Hz the relationship between CPP/ABP and CBFV is likely determined predominantly by the impedance properties of the cerebral vascular system, i.e., vascular resistance and compliance [12]. It is likely that, with increasing frequency, biophysical properties become more significant and autoregulatory processes, including neurogenic, myogenic and endothelial control, become less able to stabilize CBF in the face of changing perfusion pressure [12].

Low-frequency autoregulatory response can been tested continuously by measuring the mean velocity autoregulatory index Mx, which is a moving correlation coefficient between CPP and CBFV [10], as well as CBFV slow waves (SWs), which reflect dynamic oscillations in cerebral blood volume related to autoregulatory vasodilatation and vasoconstriction [13]. Furthermore, high-frequency components of autoregulation can be estimated by measurement of cerebrovascular resistance (CVR), compliance (Ca) and cerebrovascular time constant (tau), being a product of CVR and Ca [14–17]. An important advantage of the tau is its independence of the cross-sectional area of the insonated vessel, which enables a comparison between patients with different vessel radii [17].

Finally, complexity analysis of TCD-derived CBFV signals has been applied for assessing a possible ‘decomplexification’ of cerebral circulation during different pathologic states [18, 19].

Since a single TCD measurement of CBFV is not sufficient when vasospasm progresses from moderate to severe, as the relationship between CPP/ABP and diameter of the vessel becomes complex [9], continuous monitoring than testing of dynamic autoregulation could help clinicians understand better cerebral hemodynamics during SAH and potentially, optimize treatment.

In this respect, shortening of tau has been found in patients suffering from SAH during VS [20], whereas a few studies estimating Mx, have shown a significant association between unilateral autoregulatory failure and development of DCI [3–5].

In addition, VS has been associated with both reduced [18] and gradually increasing complexity of CBFV [19], whereas others have proposed an asymmetry index of CBFV, based on phase shift between sides of measurement, for predicting VS [21]. However, the potential relationship of these metrics with the occurrence of DCI has not been evaluated yet. Regarding SWs, their nature remains elusive since they have been observed in both healthy and pathologic states [13]. Nevertheless, their absence has been associated with worse outcome in patients with traumatic brain injury (TBI) [22].

The primary aim of this study was to find a potential pathophysiological link between VS and DCI in patients suffering from SAH, based on different dynamic CBFV-derived indices of autoregulation. In this respect, we tried to assess for the first time, how VS affects SWs of CBFV in patients admitted to the Neurosciences and Trauma Critical Care Unit (NCCU), Department of Neurosurgery at Addenbrooke’s Hospital, Cambridge, UK, since SWs have never been tested before in this context. SWs of CBFV were measured in order to estimate the potential impact of VS upon their magnitude, as well as their relative changes in patients with and without DCI. Secondly, we measured CBFV’s SampEn as a marker of its complexity, as well as both Mx and tau, which are considered surrogate markers of low and high-frequency components of cerebral autoregulation, respectively. The aim was to explore their potential changes during VS, as well as their differences between subgroups of patients with and without DCI. Finally, we explored if there is any prognostic value of different autoregulatory indices measured before spasm, related to the occurrence of DCI. Similarly with our previous studies [4, 5], we decided to choose this timeframe because we believe that it augments clinical usefulness of the present study, allowing early risk stratification and closer monitoring of patients at high risk of DCI.

## Methods

### Study population

We retrospectively analyzed digitally recorded and prospectively collected data from patients admitted to the NCCU, Department of Neurosurgery at Addenbrooke’s Hospital between June 2010 and January 2012 with a diagnosis of SAH, examined with TCD to assess state of autoregulation and detect VS [4, 5]. Written consent and approval of the study was given by both patients and the local Addenbrooke’s Research Ethics Committee, respectively.

**Fig. 1:**
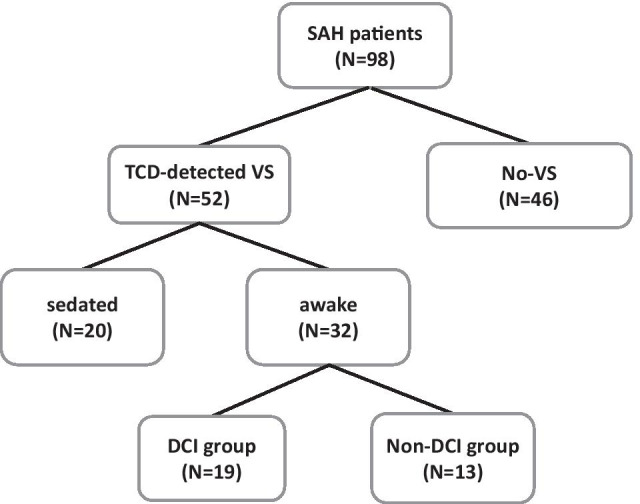
Flowchart of the study. From an initial data base of 98 patients with subarachnoid hemorrhage (SAH), 52 subjects with TCD-detected vasospasm (VS) were selected. Subsequently, 32 awake patients were included in the study and were further dichotomized in delayed cerebral ischemia (DCI) and non-DCI groups (*N* = 19 and *N* = 13, respectively)

Inclusion criteria were as follows: awake patients with ≥ 18 years of age, aneurysmal SAH confirmed with either CT or digital subtraction angiography (DSA), TCD-detected vasospasm and less than 5 days elapsed from ictus. Patients with unclear history of ictus were excluded from the study. Study design is presented in Fig. 1. Out of 98 patients included in original studies [4, 5], 66 subjects were excluded since they were either sedated (*n* = 20, 30%) or did not develop VS (*n* = 46, 70%). The remaining 32 conscious patients (mean age: 52.4 ± 10, 12 males and 20 females) who developed TCD-detected VS were further dichotomized into a DCI (*n* = 19, 59%) and a non-DCI group (*n* = 13, 41%). We decided to include only awake patients in our study in order to minimize the potential impact of sedation on different markers of cerebral autoregulation, particularly SWs of CBFV [23]. In addition, we excluded patients without VS since the primary aim of this investigation was to find a potential link between VS and DCI. Moreover, and according to the initial data base of 98 patients, from the 32 subjects with DCI only 4 did not develop VS (12.5%), limiting the possibility to detect different or similar changes of the indices measured in patients with and without VS, in relation with the occurrence of DCI (Fig. 1). Vasospasm was defined as mean blood flow velocity in the middle cerebral artery (CBFV_MCA_) higher than 120 cm/s and Lindegaard ratio (LR), which is the ratio between blood flow velocity of MCA and internal carotid artery (ICA), higher than 3 [24, 25]. Median onset of VS was 6 days after SAH, whereas DCI occurred within 21 days of ictus. DCI was defined as a drop of ≥ 2 points on the Glasgow coma scale (GCS) lasting more than 2 h, after excluding intracranial hemorrhage, acute hydrocephalus, seizures, metabolic derangements or infection, with or without radiological signs of cerebral VS, as has been previously described [4, 5, 26]. Confirmation of DCI was made through imaging in unconscious patients [4, 5].

All patients were treated with oral nimodipine 60 mg every 4 h, whereas those we developed DCI received hypertensive [27], hypervolemic and hemodilutional therapy. Neurologic status upon admission was assessed using GCS and the World Federation of Neurosurgical Societies (WFNS) scale, whereas modified Fisher scale was used for grading the amount of subarachnoid blood [28]. The Glasgow Outcome Scale (GOS) was used for assessing outcome at discharge from hospital.

### Monitoring and data analysis

Arterial blood pressure was monitored non-invasively with Finapres 2300 (Ohmeda, Amsterdam, the Netherlands) via a finger cuff, with the hand kept at the heart level in all patients. Patients were supine with the head of the bed raised 30^0^ to 45^0^. In one patient ABP was monitored invasively from the radial arterial artery using a pressure monitoring kit (Baxter HealthCare, CA).

Bilateral TCD examinations of extracranial internal carotid arteries and MCA were performed, using 2 MHz probes with Doppler Box (DWL Compumedics Germany). All patients had TCD performed every 2 days, both before (days 0 to 5 post-SAH for VS detection) and during VS, by the same operator (KB). In the present study, although screening was performed mostly during morning rounds, the monitoring times were not standardized and were subject to influence by clinical factors such as scans, relatives, etc., which potentially may account for some of the heterogeneity seen, but represents clinical reality.

The raw data signals were recorded at sampling frequency of 100 Hz using ICM + software (Cambridge Enterprise, Cambridge, UK, https://icmplus.neurosurg.cam.ac.uk/). Mean values of signals were calculated by averaging their values in a 10-s time window and then secondly averaging over the whole monitoring period (30–40 min). Only sessions with minimum of 30 min of simultaneous ABP and bilateral CBFV_MCA_ recordings were included in the analyses.

Average measurements from 3 days of TCD recordings before and during VS, as well as differences between ipsilateral and contralateral to the VS sides were compared for all patients. Both location of VS on TCD and lateralization of ischemic symptoms were used for assessing hemispheric differences of measured cerebrovascular properties. When bilateral VS was present, the analysis included averaging of both sides.

### Calculation of different cerebrovascular parameters.

#### Slow waves (SWs)

The magnitude of slow waves was assessed using spectral analysis of CBFV_MCA_ and was calculated as the square root of the power of the signal in the frequency range between 0.05 and 0.005 Hz [17], using ICM + software. Artifacts were manually removed prior to analysis.

#### Cerebral arterial time constant (tau)

The time constant of cerebral arterial bed is a TCD-derived index indicating how fast ‘arterial blood stabilizes after a change in ABP’ [17, 20]. In other words, it can reflect ‘the time of the filling arterial bed distal to the level of insonated vessel, following cardiac systole’ [17]. In that case, although CBFV_MCA_ is measured within a large artery, tau describes the distal vascular network as the product of Ca and CVRa:

1 $${\text{tau}} = {\text{Ca}}*{\text{CVRa}} = \left( {{\text{AMP}}_{{{\text{CaBV}}}} *{\text{Sa}}/{\text{AMP}}_{{{\text{ABP}}}} } \right)*\left[ {{\text{mean ABP}}/({\text{mean CBFV}}_{{{\text{MCA}}}} *{\text{ Sa}}} \right)\left] \, \right[{\text{s}}],$$

where Sa is the cross-sectional area of the insonated vessel that can be ultimately omitted from the equation, CVRa is the resistance of small cerebral arteries and arterioles estimated using ABP instead of cerebral perfusion pressure, AMP_CaBV_ and AMP_ABP_ are the fundamental harmonic amplitudes of the pulse changes of cerebral arterial blood volume (CaBV) and ABP, respectively, calculated using Fast Fourier transformation of their original time series. Pulsatile changes of cerebral arterial blood volume (ΔCaBV) can be estimated using the methodology described by Avezaat and van Eijnhoven [29], where ΔCaBV during a cardiac cycle is calculated as an integral of the difference between arterial pulsatile inflow and venous outflow of CaBV [15, 16].

#### Mean velocity autoregulation index (Mxa)

The TCD-derived mean velocity index (Mx) can be measured for assessing cerebral autoregulation. It is calculated as a Pearson’s moving correlation coefficient between 30 consecutive samples of averaged (over 10 s) cerebral perfusion pressure (CPP) and mean CBFV_MCA_ with an update every 10 s [10]. In this study, ABP instead of CPP was measured, giving rise to the Mxa index. A passive transmission of ABP fluctuations to mean flow velocity reflects impaired cerebral autoregulation and therefore, the calculated Mxa will be positive. A zero or negative Mxa signifies none or inverse association between ABP and CBFV_MCA_, something that is associated with preserved autoregulation [10]. In patients suffering from SAH, it has been suggested that values of Mxa during VS greater than 0.46 indicate impaired autoregulation [30].

#### Sample entropy (SampEn)

We applied SampEn as a measure of complexity of physiological time series [31]. Sample entropy represents the negative natural logarithm of the conditional probability that two sequences similar for *m* points remain similar at the next point (*m* + 1) with a tolerance r. The parameter r that is the tolerance for accepting matches, is usually set between 15 and 25% of standard deviation (SD) of the time series after normalization (SD = 1). The parameter m (embedding dimension) is the length of sequences to be compared and its values is usually set to 1 or 2 for data length ranging from 100 to 5000 data points. In our analysis, we computed SampEn of CBFV_MCA_ signals, using ICM + software assigning the values of 2 for *m* and 0.15 for *r*.

### Statistical analysis

Statistical analysis was performed using IBM SPSS Statistics 26 package (Armonk, NY, USA). Patients were dichotomized into DCI and non-DCI groups. The assumption of normal distribution was confirmed using Shapiro–Wilk test, at the significance level of 0.05. Therefore, parametric tests were used. Age and ABP were compared between groups with 2-tailed *t* test, whereas ABP values between times of measurements with paired *t* test, respectively. The Fisher’s exact test was applied for categorical variables.

A two-way analysis of variance (ANOVA) was performed to detect the potential effects of time and side of measurements on CBFV-derived variables for the whole group of patients. In addition, a two-way mixed-design ANOVA was applied for assessing temporal differences between and within groups, as well as their potential interactions. Homogeneity of variance was assessed with Levene’s test, whereas Sidak adjustment was applied for multiple comparisons. Sidak adjusted *p* values were measured using the formula: *p*(adjusted) = 1–[1–*p* (unadjusted)]^number of pairs^ and were compared with the significance level of 0.05 [32]. Since we measured 5 variables (CBFV, CBFV SWs, tau, Mxa and SampEn) at two different time points and side of measurements in the whole studying population, the number of pairs was 20. The same level of significance was adopted for comparisons between and within groups (ipsilateral to VS side before and during spasm for each group). Values were averaged per monitoring session, before and during VS, based on the TCD onset of VS for each patient.

Bivariate correlations between the five, ipsilateral to VS side, measured variables, before and during spasm in both groups, were estimated using the Pearson *r* coefficient. Due to multiple comparisons and in order to protect from type 1 error (false positives), a post hoc Sidak correction was applied.

A binary multiple logistic regression model was used to assess the ability of CBFV-derived autoregulatory indices on days 0 to 5 post-ictus to predict the development of DCI. Thus, using the forward stepwise regression method we run the univariate analyses, relating each predictor with the outcome of interest one at a time and then, we run a multivariable model. Finally, receiver operating characteristic (ROC) curve for predicting DCI was constructed. Data are presented as mean ± SD, whereas significance level was set at *α* = 0.05.

## Results

Table 1 summarizes the baseline characteristics for the included patients divided by the presence of DCI. The two cohorts did not differ in terms of age, WFNS and modified Fisher scales, GCS upon both admission and discharge, as well as GOS. ABP values did not differ significantly between pre-VS and VS period of measurements, neither for the whole population nor between groups.

**Table 1:** Characteristics of all patients and subgroups with and without DCI

Variables	Overall	DCI	Non-DCI	*p* values
	(*N* = 32)	(*N* = 19)	(*N* = 13)	
Age, years ± SD	52.4 ± 10	54.15 ± 11.8	50 ± 7.6	0.27^$^
Sex (male/female)	12/20	8/11	4/9	0.74^#^
WFNS (mean)	2.37 ± 1.33	2.36 ± 1.38	2.38 ± 1.32	0.97^#^
Modified Fisher scale	3 ± 0.8	3.2 ± 0.7	3.1 ± 1	0.94^#^
GCS admission	12.2 ± 3.4	12.4 ± 3.2	11.9 ± 3.8	0.70^#^
GCS discharge	13.8 ± 2.9	13.2 ± 3	14.1 ± 1	0.16^#^
GOS	4 ± 1	4 ± 1	4 ± 1	0.55^#^
Aneurysm location				
*1. AcomA*	9	6	3	0.53^#^
*2. MCA*	8	4	4	0.93^#^
*3. PcomA*	9	6	3	0.12^#^
*4. PICA*	4	2	2	0.46^#^
*5. BA*	1	0	1	0.35^#^
*6. ICA*	1	1	0	0.32^#^
VS side				
*1. Right*	14	9	5	0.44^#^
*2. Left*	11	6	5	0.67^#^
*3. Bilateral*	7	4	3	0.72^#^
Clipping/coiling	20/12	12/7	8/5	0.40^#^
Rebleeding	1	0	1	0.32^#^

*DCI* delayed cerebral ischemia, *WFNS* World Federation of Neurosurgical Societies, *GCS* Glasgow Coma Scale, *GOS* Glasgow Outcome Scale, *AcomA* anterior communicating artery, *MCA* middle cerebral artery, *PcomA* posterior communicating artery, *PICA* posterior inferior cerebellar artery, *BA* basilar artery, *ICA* internal carotid artery, *VS* vasospasm, *SD* standard deviation

^$^2-tailed *t*-test, ^#^ Fisher’s exact test

Tables 2 and 3 present differences of measured TCD-derived variables for all patients, as well as between and within groups, respectively.

**Table 2:** Temporal and spatial differences of measured variables across the overall study population

Variables	Side of measurement	Time of measurement (pre-VS)	Time of measurement (during VS)	*p* value
CBFV (cm/s)	Ipsilateral	89.6 ± 22.5	156.2 ± 23.7	** < 0.001**
	Contralateral	78.9 ± 31.4	100.5 ± 43.7	0.92
CBFV SWs	*p* value	0.94	0.17	
[cm/s]	Ipsilateral	2.86 ± 1.21	4.15 ± 1.55	** < 0.001**
	Contralateral	2.89 ± 1.48	3.29 ± 1.30	0.95
Tau	*p* value	0.92	0.17	
[s]	Ipsilateral	0.25 ± 0.17	0.17 ± 0.08	0.19
	Contralateral	0.28 ± 0.16	0.19 ± 0.09	0.83
Mxa	*p* value	0.98	0.97	
	Ipsilateral	0.24 ± 0.20	0.31 ± 0.21	0.16
	Contralateral	0.20 ± 0.18	0.23 ± 0.26	0.92
SampEn	*p* value	0.95	0.8	
	Ipsilateral	2.67 ± 0.93	3.00 ± 0.96	0.83
	Contralateral	2.95 ± 1.32	3.16 ± 2.15	0.83
	*p* value	0.95	0.95	

*N* = 32, two-way ANOVA, post hoc comparisons, SPSS Sidak adjusted *p* values

*VS* vasospasm, *Tau* time constant, *CBFV* cerebral blood flow velocity, *SWs* slow waves, *Mxa* mean autoregulatory velocity index, *SampEn* sample entropy

**Table 3:** Temporal differences of measured variables ipsilateral to VS side, between and within groups (two-way mixed-design ANOVA, post hoc comparisons, SPSS Sidak adjusted *p* values)

Variables	Time of measurement	DCI (*N* = 19)	Non-DCI (*N* = 13)	*p* value
CBFV (cm/s)	Pre VS	90.9 ± 25.5	85.9 ± 22.4	0.93
	During VS	150.8 ± 33.2	167.9 ± 33.7	0.9
	*p* value	** < 0.001**	** < 0.001**	
CBFV SWs [cm/s]	Pre VS	3.20 ± 1.02	2.25 ± 1.08	** < 0.001**
	During VS	3.95 ± 1.45	4.64 ± 1.43	0.91
	*p* value	0.84	** < 0.001**	
Tau (s)	Pre VS	0.31 ± 0.20	0.20 ± 0.09	0.9
	During VS	0.18 ± 0.09	0.17 ± 0.10	0.97
	*p* value	0.71	0.93	
Mxa	Pre VS	0.23 ± 0.23	0.26 ± 0.16	0.95
	During VS	0.36 ± 0.18	0.26 ± 0.23	0.85
	*p* value	0.85	0.93	
SampEn	Pre VS	2.60 ± 0.90	2.70 ± 0.90	0.9
	During VS	3.03 ± 1.01	2.90 ± 0.90	0.95
	*p* value	0.9	0.95	

*VS* vasospasm, *DCI* delayed cerebral ischemia, *tau* time constant, *CBFV* cerebral blood flow velocity, *SWs* slow waves, *Mxa* mean autoregulatory velocity index, *SampEn* sample entropy

Ipsilateral mean CBFV values were significantly increased during VS in all patients (spasm: 156.2 ± 23.7 vs before: 89.6 ± 22.5 cm/s, *p* < 0.001, Table 2), as well as within groups (spasm: 150.8 ± 33.2 vs before: 90.9 ± 25.5 cm/s, *p* < 0.001 for DCI and 167.9 ± 33.7 vs 85.9 ± 22.4 cm/s, *p* < 0.001 for non-DCI group, respectively, Table 3). However, temporal differences were not significant between groups.

### Slow waves of CBFV

During VS, magnitude of SWs of CBFV was significantly higher related to pre-VS measurements on the ipsilateral side (4.15 ± 1.55 vs 2.86 ± 1.21 cm/s, *p* < 0.001, 95% CI: 0.84–1.9, Table 2) for the whole studying population. Similar differences were found during VS between spatial assessments without reaching statistical significance.

**Fig. 2:**
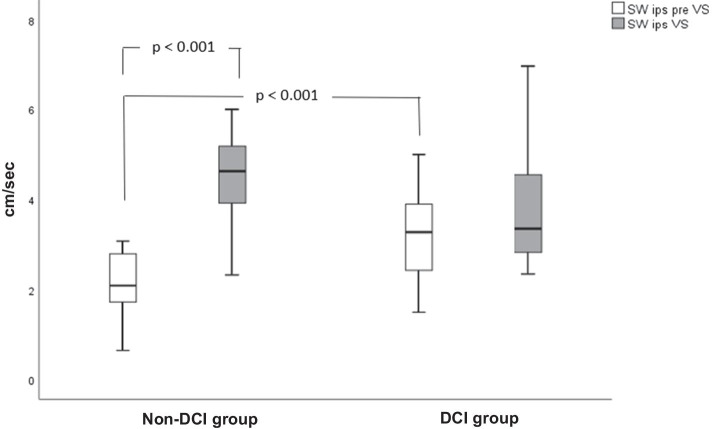
Statistically significant temporal differences of ipsilateral (ips) to VS side CBFV SWs are illustrated between and within delayed cerebral ischemia (DCI) and non-DCI groups. Units of measurement in the *y* axis are cm/s

Ipsilateral CBFV SWs were significantly higher during VS in the non-DCI group compared to pre-VS values (4.64 ± 1.43 vs 2.25 ± 1.08 cm/s, *p* < 0.001, 95% CI: 1.63–2.82, Table 3, Fig. 2). Moreover, patients with DCI had significantly higher ipsilateral SWs before VS in relation with non-DCI group (3.20 ± 1.02 vs 2.25 ± 1.08 cm/s, *p* < 0.001, 95% CI: 0.28–1.73, Table 3).

### Tau, Mxa and SampEn.

Ipsilateral tau during VS was shortened in relation to pre-VS period of measurements in the whole studying population but without reaching statistical significance. Furthermore, ipsilateral differences were not significant between groups, either before or during VS (Table 3).

Ipsilateral Mxa was increased during VS in DCI vs non-DCI groups of patients, as well as within the DCI group but non-significantly.

Moreover, ipsilateral SampEn was higher during VS compared to pre-VS values but differences were not statistically significant (Table 2). Similarly with tau, SampEn did not differ between groups during different times of measurements. Finally, no significant interactions were found between measured variables.

### Bivariate correlations

No significant correlations were found between measured variables before and during spasm, except for ipsilateral pre-VS mean CBFV, which was positively correlated to the magnitude of CBFV SWs in the DCI group (*r* = 0.7, *p* < 0.001).

### Prediction of DCI

**Fig. 3:**
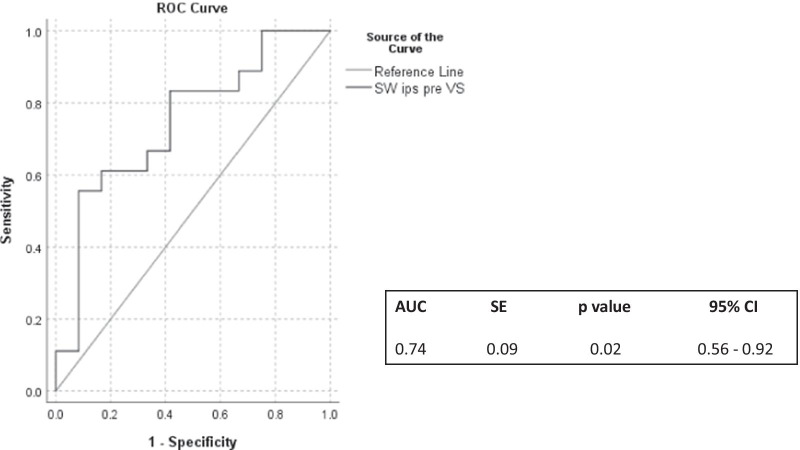
Receiver operating characteristic (ROC) curve with *p* value of area under the curve (AUC), standard error (SE) and 95% confidence intervals (CI) of ipsilateral CBFV SWs, measured before VS (SW ips pre-VS), for predicting delayed cerebral ischemia (DCI)

When a binary logistic regression model with pre-VS values of CBFV, CBFV SWs, Mxa and tau was used, only CBFV SWs remained significant predictors of DCI with odds ratio (OR) 2.52 (95% CI: 1.05–6.02) and standard error (SE) 0.44. The model showed that one-unit change in pre-VS SWs resulted in a 0.92-unit change in the log of the OR. ROC curve analysis found an AUC of 0.745 with 95% CI between 0.56 and 0.92 and SE 0.094 (*p* = 0.02, Fig. 3). Furthermore, SWs’ cut-off value of 2.8 was able to predict DCI with a balanced sensitivity and specificity of around 60%.

## Discussion

In the present study, we evaluated how a hemodynamic insult such as VS, might affect CBFV’s SWs and SampEn, as well as Mxa and tau values, in patients suffering from SAH, dichotomized by the presence of DCI. We also hypothesized that a particular combination of measured metrics might predict DCI, constituting a novel cluster of ‘physiomarkers’ for monitoring of cerebral autoregulation in the ICU setting. Such markers could also be promising tools for early therapeutic intervention in vulnerable patients, even before clinical or TCD-derived signs of VS appear [20].

### Temporal differences for the whole group of patients

For the whole studying population, we found that during VS, ipsilateral CBFV SWs were significantly higher in relation to pre-VS measurements. Despite lack of statistical significance of our findings, we also confirm previous study by Kasprowicz and colleagues [20], who found that tau was reduced during VS even before formal TCD signs of VS were observed.

CBFV SWs with an associated frequency range of 0.005 to 0.05 Hz, reflect dynamic oscillations in cerebral blood volume related to autoregulatory vasodilatation and vasoconstriction [13]. Fluctuations of CBFV measured with TCD have been found to occur simultaneously with intracranial pressure (ICP) B-waves and occupy the same frequency [13, 22].

The physiological and clinical significance of SWs remains debated since they do not only occur during pathologic conditions but have also been observed in healthy subjects [13]. Different theories of origin have been developed, relating SWs with pCO_2_ changes-induced oscillations in the cerebrovascular volume [33], plateau waves of ICP waveforms [34], or rhythmic cerebral vasoconstriction caused by an intrinsic brain stem rhythm [35]. In this respect, general anesthesia has been found to reduce amplitude of SWs of ICP [23]. Moreover, Greitz and colleagues [36], have proposed that restricted arterial distensibility due to decreased intracranial compliance is associated with increased capillary pulsations and subsequently, higher CBFV SWs.

According to classic studies of Fry and Byrom [37, 38], when cerebral arteries are narrowed, like in cases of VS, flow pulsations and wall shear stress will be enhanced, tending to increase the power dissipation and thus, the pressure gradient along the vascular tree. In this case, all sequential branches of the capillary network try to dilate in order to avoid or decrease this augmented pressure drop, through production of local vasodilatory molecules, such as nitric oxide (NO). Such metabolic effects might be reflected in the amplitude of blood flow oscillations within a frequency range below 0.05 Hz, as has been shown by Stefanovska and colleagues in peripheral blood flow [39] and described in the frequency-dependent behavior of cerebral autoregulation [12].

Based on the previous discussion, we suggest that higher CBFV SWs during VS correspond to local mechanisms related to increased capillary stress. In this case, stiffening of large conduit arteries due to VS might induce increased and faster pressure and volume transmission into the brain capillaries. Although VS is mainly associated with vessel narrowing, where volume transmission is not necessarily increased, we suppose that shortened tau (even non-significantly) might reflect acceleration of the volume transmission, which in association with increased blood flow rate might enhance wall shear stress in the capillary network distal to insonation site. Consequently, such effects could increase amplitude of oscillations below 0.05 Hz due to enhanced production of different vasodilatory molecules, as an endothelial response to augmented shear stress. In addition, lack of significant correlation between ipsilateral mean CBFV and CBFV SWs might reflect their dissociation due to vessel lumen narrowing during VS. Finally, triple H therapy cannot account for our results, since ABP values did not differ significantly between pre-VS and VS period of measurements.

Cerebral circulation can be considered as a complex system, since it involves both central and peripheral control mechanisms through multiple feedback loops [40, 41]. Decreased complexity reflects either decreased information content or decreased disorder, related to the number of ‘microstates’ that are accessible to the system [40].

Soehle and colleagues [18], evaluated complexity of CBFV signals in patients suffering from SAH and found significantly reduced values during VS. On the contrary, Placek and coworkers [19], found that VS was associated with gradually increasing complexity of CBFV, attributed to a potential improvement in autoregulation and the number of regulatory mechanisms involved with its variability. Additionally, complexity of blood flow was significantly reduced ipsilateral to aneurysm rupture related to contralateral side before occurrence of VS, suggesting a potential therapeutic window.

In our investigation and similarly with Placek’s study [19], SampEn was also reduced ipsilateral compared to contralateral side, both before and during VS, but without reaching statistical significance. We suggest that differences between methods for assessing complexity could be responsible for inconsistency across different studies.

### Temporal differences between and within DCI and non-DCI groups

Ipsilateral Mxa values were increased during VS in patients with DCI related to pre-VS measurements, as well as in DCI compared to non-DCI group. Nevertheless, differences were not significant. Such findings are similar with previous work from different research groups [3–5].

Ipsilateral CBFV SWs before VS were significantly higher in the DCI compared to the non-DCI cohort. Moreover, non-DCI patients exhibited significantly higher SWs during VS related to pre spasm values (Fig. 2). Since there are no similar studies in the literature, we can only guess about the potential pathophysiological mechanisms of such findings. Thus, we suggest that patients with DCI might experience an increased capillary stress even before the occurrence of VS. Since the two groups did not differ in terms of pre-VS values of Mxa, we suppose that the increased heterogeneity of flow during the first days after SAH that has been found in a few studies [42], might reflect focal impairment of autoregulation, which cannot be captured with TCD-derived indices. In this respect, a positive correlation was found between mean CBFV and SWs before VS in the DCI group, suggesting an association between SWs and fluctuations in CBF.

Furthermore, higher pre-VS SWs in the DCI group could also be attributed to enhanced local neurogenic inputs originating in the brain stem, which are independent of the sympathetic nervous system [43]. It has been suggested that in cases of highly focal disturbances of flow and autoregulation, inputs from the brain stem towards cerebral blood vessels are increased, in order to preserve CBF at the level of microcirculation [43]. In such cases, magnitude of CBFV SWs will be augmented, since different local neurogenic mechanisms are also considered responsible for blood flow oscillations within a frequency range below 0.05 Hz [12, 39]. Thus, the association of pre-VS SWs with occurrence of DCI that was found in the regression analysis might reflect the positive predictive value of regional dysautoregulation. In addition, lack of significant collinearity between input variables in the regression model due to absence of any significant correlation between them, increases precision of our findings. Nevertheless, these results need to be validated in a larger prospective study.

Finally, modest increase in CBFV SWs during VS in DCI patients related to non-DCI group, could be related to a state of vasoparalysis, since such condition may partially account for a reduction in blood vessels oscillatory capacity. It seems that a decreased vasomotor tone due to dysautoregulation, reflected in increased Mxa values (even non-significant), is associated with loss of pressure reactivity of cerebral blood vessels, limiting the ability of endothelial factors to induce oscillations in vessels’ wall, with subsequent attenuated increase in the amplitude of CBFV SWs. In this respect, different experimental studies have found that cortical arterioles after VS due to SAH demonstrate attenuated dilation to different endothelial-dependent dilators [44], reflecting microvascular endothelial dysfunction.

### Strengths and limitations of the study

Some of the major limitations of this study is its retrospective nature, as well as the small sample size. Nevertheless, we included only conscious patients, in order to have clinically proven diagnosis of DCI, since its diagnosis in sedated subjects is more difficult and depends on different imaging techniques [27]. Moreover, the mixture of both sedated and conscious patients might dilute findings in terms of SWs changes [23].

Mean CBFV did not differ between groups significantly, both before and during spasm. Thus, our patients seem to have similar severity of VS, limiting its potential impact on our findings.

Measurement of ABP rather than CPP for calculation of Mx might limit accuracy of our results. However, both Mx and Mxa have been shown to exhibit good correlation, particularly in cases of impaired autoregulation in TBI patients [45].

Another potential confounder in our study might be the arterial tension of pCO_2_, limiting accuracy of comparisons between patients [11]. Nevertheless, none from our patients had a history of chronic obstructive pulmonary disease or any other pulmonary disease that might affect pCO_2_ levels, assuming that its potential impact upon our measurements might be insignificant.

Non-invasive ABP measurement for Mx calculation through Finapres system could constitute a further limitation in terms of accuracy of results. However, agreement between invasive and non-invasive assessment of Mx has been tested and a good correlation between the two methods was found [46].

Finally, lack of a normal control group constitutes another limitation of our study. Thus, lack of normative values of SWs does not permit us to define accurately their change between and within groups. Moreover, the inclusion of patients without VS might shed more light into potential pathophysiological mechanisms associated with the occurrence of DCI, in case of different or similar changes found between SAH patients with and without VS. Nevertheless, this can be the objective of a new prospective study.

In conclusion, we suggest that daily monitoring of CBFV SWs using online processing systems that support real-time processing of multiple high-rate physiological data streams, might have added value in the ICU, supporting clinical decisions at the bedside [47]. Thus, longitudinal changes of SWs, even in the early days post-ictus, could help identify patients who are more susceptible to development of DCI throughout their hospital course and prompt early treatment in a proactive rather than a reactive way. Consequently, such methods might determine the potential for early risk stratification and probably closer observation in the ICU for patients at high risk of DCI.

## Conclusions

Slow waves of CBFV in DCI group were significantly higher before VS and predicted unfavorable outcome. Consequently, we suggest that following SAH, their daily monitoring through TCD measurements at the bedside could determine the potential for early risk stratification and eventually, optimize therapeutic management through early escalation of treatment.

## Data Availability

The datasets used and analyzed during the current study are available from the corresponding author on reasonable request.

## Abbreviations

ABP: Arterial blood pressure
AMP: Amplitude
ANOVA: Analysis of variance
AUC: Area under the curve
CBF: Cerebral blood flow
CBFV: Cerebral blood flow velocity
Ca: Compliance
CI: Confidence intervals
CPP: Cerebral perfusion pressure
CVR: Cerebral vascular resistance
DCI: Delayed cerebral ischemia
GSC: Glasgow Coma Scale
GOS: Glasgow Outcome Scale
ICA: Internal carotid artery
ICP: Intracranial pressure
LR: Lindegaard ratio
MCA: Middle cerebral artery
Mxa: Mean velocity autoregulatory index
OR: Odds ratio
ROC: Receiver operating characteristic
SAH: Subarachnoid hemorrhage
SampEn: Sample entropy
SWs: Slow waves
tau: Time constant
TCD: Transcranial Doppler
VS: Vasospasm
WFNS: World Federation of Neurosurgical Societies Scale
